# Capillary-Driven Microfluidic Electrical Screening of Influenza H3N2-Infected A549 Cells Using AgNP-Decorated Laser-Patterned Villous Microstructures

**DOI:** 10.3390/bios16070375

**Published:** 2026-07-09

**Authors:** Zhaochi Chen, Minh-Quang Tran

**Affiliations:** 1 Graduate Institute of Biomedical Optomechatronics, Taipei Medical University, Taipei 230, Taiwan; 2 Department of Mechanical Engineering, TUETECH University, Thai Nguyen 250000, Vietnam

**Keywords:** capillary-driven microfluidics, AgNPs-decorated villous microstructures, H3N2-infected A549 cells, label-free electrical screening

## Abstract

A capillary-driven microfluidic electrical screening platform was developed using silver nanoparticle (AgNP)-decorated laser-patterned villous microstructures on a glass substrate for the analysis of H3N2-infected A549 cells. The device integrated nanosecond laser patterning, AgNP conductive thin-film formation, passive capillary transport, and direct electrical readout within a single microfluidic sensing structure. Villous-like arrays were fabricated using a 1064 nm IR pulsed laser at a fluence of 4.35 J/cm^2^, with a repetition rate of 300 kHz, pulse overlap of 96.7% and scanning speed of 500 mm/s. The fabricated structures exhibited a diameter of 60 μm, height of 80 μm and interpillar pitches ranging from 30 to 90 μm. After AgNP deposition, the surface showed a dominant Ag content of 59.2%, confirming successful formation of conductive microstructured electrodes. The 30 μm pitch structure produced the highest current response of 22 μA at 1 V and the highest Δ*I_norm_* of 0.053 after introduction of H3N2-infected A549 samples. Wettability and capillary transport were tunable by pitch, with contact angles (CAs) decreasing from 140° to 30° and flow velocities decreasing from 0.1 mm/s to 0.03 mm/s. Formalin-fixed H3N2-infected A549 cells were electrically distinguished from non-infected A549 controls over 10^1^–10^6^ PFU/μL, with detectable responses down to 10^1^ PFU/μL. These results demonstrate a label-free, self-driven, and fabrication-oriented microfluidic strategy for electrical screening of virus-associated cellular samples.

## 1. Introduction

Influenza A virus remains a major cause of acute respiratory infection and continues to impose a substantial burden on public health [1,2]. In addition to seasonal outbreaks, the rapid evolution of influenza viruses and the overlap of their clinical symptoms with other respiratory diseases place strong pressure on diagnostic systems to provide timely [3,4], accurate [5], and scalable testing [6]. Recent studies have therefore emphasized the need for biosensing strategies that can complement conventional virological and molecular assays with shorter turnaround time [7], lower operational complexity [8], and improved suitability for decentralized settings [9,10]. While nucleic-acid-based methods remain the benchmark for sensitivity, they often require trained personnel, dedicated instrumentation, and multistep sample handling, which can limit rapid deployment in point-of-care and field-use scenarios [11,12]. Beyond direct viral identification, influenza infection induces host-cell responses including membrane remodeling, cytopathic alterations, and changes in cellular electrical characteristics, creating opportunities for label-free electrical screening approaches capable of distinguishing infected cells from healthy counterparts.

To address these limitations, biosensors and microfluidic analytical systems have attracted increasing attention because they enable miniaturized sample processing [13], reduced reagent consumption [14], and integrated signal readout [15]. In particular, recent progress in infectious-disease biosensing has highlighted the value of electrochemical, optical, and nucleic-acid-responsive platforms for rapid pathogen analysis [16,17]. For influenza-related diagnostics, representative advances include electrochemical amplification schemes, SERS-based viral sensing [18], and microfluidic LAMP-enabled assays that support multiplex or near-point-of-care detection [19,20]. However, many high-performance systems still rely on external pumping, complex reagent choreography, or molecular amplification workflows that increase device complexity and reduce practical portability [21,22].

Capillary-driven microfluidics offers an attractive route toward self-driven bioanalysis because passive fluid transport can be encoded directly into channel geometry, interfacial chemistry, and microstructured surface design [23,24]. Recent studies have shown that 3D manufacturing strategies, including engineered capillary valves, paper-based flow networks, and hollow-channel microfluidics, can provide programmable liquid transport without bulky peripheral equipment [25,26]. Doloi et al. demonstrated that AI-integrated self-driving laboratory platforms enable autonomous experimental optimization, achieving over 24,000 sequential experimental operations within 329 h, thereby significantly enhancing experimental throughput and reproducibility in materials discovery workflows [27]. Lee et al. reported that 3D-printed droplet microfluidic devices enable deterministic droplet generation governed by key dimensionless parameters, including capillary number, viscosity ratio, and flow-rate ratio, producing highly uniform droplets with characteristic diameters of approximately 20–200 μm and low coefficient of variation [28]. Such features are particularly advantageous for rapid screening platforms, where device simplicity, reproducibility, low cost, and autonomous liquid transport are essential [29]. Importantly, the coupling of capillary microfluidics with biosensing modules has enabled integrated diagnostic devices for infection screening, environmental monitoring, and portable analytical testing [30,31].

Among substrate materials for microfluidic device fabrication, glass remains highly attractive because of its chemical inertness, thermal stability, electrical insulation, optical transparency, and compatibility with surface modification [32]. These properties are valuable for microfluidic biosensors that require robust channel architecture and stable sensing interfaces [33]. Nevertheless, conventional fabrication strategies for glass microdevices often involve multistep lithographic or etching workflows that are difficult to scale efficiently for complex multiscale structures [34]. Recent work has therefore focused on laser-enabled glass processing, which provides maskless, localized, and high-resolution fabrication of three-dimensional microchannels and functional surface textures [16,35]. Chen et al. developed a laser-fabricated glass-based capillary microfluidic PCR device integrating graphene microheaters, enabling precise thermal cycling and stable capillary-driven flow through laser-defined microchannels and micropillar structures. The study demonstrated the importance of laser-patterned glass substrates for highly controlled biochemical microfluidic systems requiring chemical stability, thermal robustness, and biocompatibility [36]. Hashemi et al. demonstrated a microfluidic sensing platform incorporating laser-synthesized biochar carbon nanoparticles, enabling highly sensitive piezoresistive detection with rapid response and recovery characteristics. The results highlight the potential of laser-processed functional nanomaterials integrated into microfluidic architectures for sensitive signal transduction and real-time physiological monitoring [37]. Laser processing is particularly attractive for microfluidic biosensors because it enables direct fabrication of hierarchical villous-like microstructures that can simultaneously regulate wettability, capillary transport, sample retention, and the interfacial integration of conductive or plasmonic nanomaterials [38].

In parallel, silver nanostructures remain attractive for biosensing because they provide high electrical conductivity, rich interfacial chemistry, and compatibility with micro- and nanostructured sensing surfaces [39]. Integrating AgNP-decorated conductive layers with laser-patterned glass can therefore create multifunctional microfluidic substrates in which capillary transport regulation and electrical signal transduction are co-designed within the same platform [40]. Kumar et al. fabricated monolithic multilayer microfluidic devices via additive manufacturing, enabling rapid synthesis of AgNPs and significantly enhancing sensing sensitivity through nanoparticle-assisted signal amplification [41]. Moulahoum developed a laser-printed μPAD integrated with AgNPs for microfluidic biosensing, demonstrating the feasibility of combining laser-fabricated microfluidics with nanoparticle-enhanced sensing interfaces [42]. These studies collectively suggest that AgNP-assisted conductive microstructures can provide an effective strategy for improving electrical response sensitivity while maintaining fabrication simplicity and device portability.

Based on these considerations, this study develops a capillary-driven microfluidic electrical screening platform based on AgNP-decorated laser-patterned villous microstructures on glass for the analysis of H3N2-infected A549 cells. Rather than relying on fluorescence labeling or multistep molecular amplification, the proposed platform integrates capillary force-regulated transport, villous microstructure-assisted sample manipulation, and conductive thin-film electrical readout within a single chip architecture. By tailoring the dimensions of the laser-patterned villous microstructures, both capillary behavior and electrical responses can be modulated to enhance sample sensing interactions. Using formalin-fixed H3N2-infected A549 cells with different viral exposure levels, the proposed platform evaluates concentration-dependent electrical responses and demonstrates a fabrication-oriented strategy for simple, label-free, and self-driven screening of virus-associated cellular samples.

## 2. Materials and Methods

### 2.1. Materials and Equipment

Human lung epithelial A549 cell samples associated with influenza A virus subtype H3N2 exposure were obtained from the National Health Research Institutes (NHRI, Taiwan) and used as representative virus-associated cellular samples throughout this study. Briefly, A549 cells (BCRC 60074, Bioresource Collection and Research Center, Hsinchu, Taiwan) were cultured in Dulbecco’s Modified Eagle Medium (DMEM, Gibco, Thermo Fisher Scientific, Waltham, MA, USA) supplemented with 10% fetal bovine serum (FBS, Gibco, Thermo Fisher Scientific, Waltham, MA, USA) and 1% antibiotic–antimycotic solution under standard cell-culture conditions. The cultured A549 cells were subsequently exposed to influenza A virus subtype H3N2 and processed by NHRI to establish cellular samples with different viral exposure levels. Following sample preparation, the cells were fixed using 10% neutral buffered formalin and supplied as formalin-fixed H3N2-infected A549 cell samples for subsequent microfluidic electrical screening experiments. Phosphate-buffered saline (PBS, pH 7.4, Gibco, Thermo Fisher Scientific, Waltham, MA, USA) was used for washing, dilution, and sample preparation procedures. A 10% neutral buffered formalin solution (Sigma-Aldrich, St. Louis, MO, USA) was used for cell fixation. Cell culture was maintained in a humidified CO_2_ incubator (Thermo Fisher Scientific, Waltham, MA, USA) at 37 °C and 5% CO_2_. Soda-lime glass substrates (Ruilong Co., Ltd., Houlong, Miaoli, Taiwan) with a thickness of 1.1 mm were obtained and used as the substrate material for fabrication of the capillary-driven microfluidic devices. Laser patterning was performed using a programmable nanosecond laser processing system (IPG Photonics Co., Oxford, MA, USA) operating at a wavelength of 1064 nm. Silver nanoparticles (AgNPs, Product No. 730815, Sigma-Aldrich, Merck KGaA, Darmstadt, Germany) with an average particle diameter of 60 nm were used as the conductive coating material. The nanoparticles were supplied as a citrate-stabilized aqueous suspension with a concentration of 20 mg/mL. Sodium dodecyl sulfate (SDS, CAS No. 151-21-3, Aencore Chemical Pty. Ltd., Box Hill, VIC, Australia) was employed as a dispersing agent for preparation of AgNPs suspension. Polydimethylsiloxane (PDMS, Sylgard 184, Dow Corning, Midland, MI, USA) was used as the sealing layer of the microfluidic device. Delayed-tack adhesive tape was utilized as both the temporary masking layer and bonding interface during device fabrication. Thermal drying was performed using a convection oven (DFO-36, Yihder Co., Ltd., Zhonghe, New Taipei City, Taiwan). Surface morphology and structural characteristics of the fabricated villous microstructures were characterized using laser scanning confocal microscopy (LSCM, TCS SP8 X, Leica Microsystems GmbH, Wetzlar, Germany) and scanning electron microscopy (SEM, Helios NanoLab 1200+, FEI, Hillsboro, OR, USA). Electrical measurements was performed using a source meter (Keithley 2450, Keithley Instruments LLC, Solon, OH, USA) under a constant bias voltage of 1 V.

### 2.2. Design and Fabrication of Capillary-Driven Microfluidic Device

**Figure 1:**
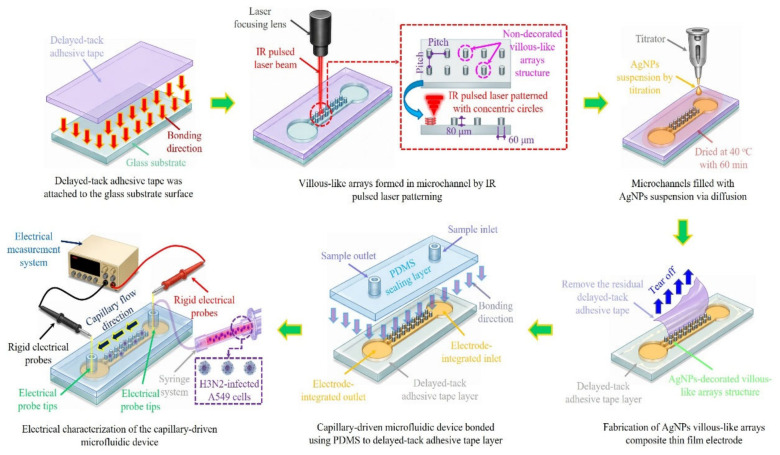
Fabrication and operation of the capillary-driven microfluidic electrical screening platform using AgNP-decorated laser-patterned villous-like electrodes for H3N2-infected A549 cell detection.

The overall fabrication process of the capillary-driven microfluidic electrical screening platform is illustrated in Figure 1. The device consisted of three functional layers, including a glass substrate, AgNP-decorated laser-patterned villous microstructures, and a PDMS sealing layer. Soda-lime glass substrate was cut into dimensions of 40 mm × 20 mm and sequentially cleaned using acetone, isopropanol, and deionized water (DI water) under ultrasonic agitation for 10 min in each solvent. After drying, a delayed-tack adhesive tape layer was laminated onto the glass surface to define the microchannel region and provide a temporary masking layer during laser processing. Microchannels, inlet and outlet reservoirs, and villous-like microstructure arrays were subsequently fabricated using programmable nanosecond laser patterning. The villous-like arrays were positioned within the sensing region of the microchannel and served as both capillary-regulating structures and conductive sensing interfaces after AgNPs decoration. The microchannel connected the sample inlet and outlet reservoirs, enabling spontaneous fluid transport driven solely by capillary force. Following laser processing, AgNPs suspension was introduced into the patterned microchannel through titration-assisted diffusion. The suspension infiltrated the villous-like arrays region and formed a conductive coating after drying at 40 °C for 60 min. The residual delayed-tack adhesive tape was subsequently removed, leaving AgNP-decorated villous-like arrays on the glass substrate. Finally, a PDMS layer containing aligned inlet and outlet openings was bonded onto the substrate to form a closed capillary-driven microfluidic device. Electrical probing pads located at both ends of the sensing region enabled real-time electrical characterization during sample transport.

### 2.3. Fabrication of AgNP-Decorated Laser-Patterned Villous Microstructures

Laser-patterned villous-like microstructure arrays were fabricated using a programmable nanosecond laser micromachining system operating at a wavelength of 1064 nm. The IR pulsed laser beam was delivered through a galvanometric scanning system and focused by an *F-θ* lens with a focal length of 160 mm. The focused spot diameter was approximately 50 μm, and the pulse duration was 200 ns. To generate villous-like arrays, concentric-circle scanning trajectories were employed to induce localized surface modification and controlled material removal on the glass substrate. The laser fluence was optimized to 4.35 J/cm^2^, while the repetition rate, pulse overlap ratio, and scanning speed were set at 300 kHz, 96.7%, and 500 mm/s, respectively. The fabricated villous-like arrays exhibited an average diameter of 60 μm, a height of 80 μm, and an interpillar pitch ranging from 30 μm to 90 μm. These structural parameters were selected to enhance capillary transport behavior and increase the effective electrode–sample interaction area. For conductive electrode formation, AgNPs suspension was prepared by mixing 0.5 g of AgNPs, 0.005 g of SDS, and 4.495 g of DI water, followed by magnetic stirring at 60 °C for 120 min. The resulting suspension was deposited into the laser-patterned region and allowed to diffuse throughout the villous microstructures. After drying at 40 °C for 60 min, a continuous AgNPs conductive thin-film layer was formed on the villous-like arrays, thereby forming AgNP-decorated laser-patterned villous electrodes for integration into the capillary-driven microfluidic device.

### 2.4. Preparation of H3N2-Infected A549 Cell Samples

**Figure 2:**
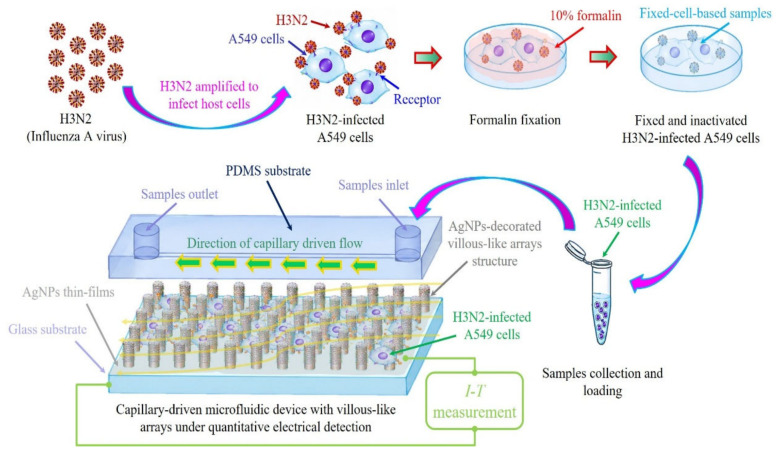
Preparation of formalin-fixed H3N2-infected A549 cells and their electrical screening in the capillary-driven microfluidic device with AgNP-decorated villous-like arrays.

The preparation procedure of fixed-cell-based samples is illustrated in Figure 2. Formalin-fixed H3N2-infected A549 cell samples were supplied by the NHRI. Briefly, A549 cells were cultured in DMEM supplemented with 10% FBS and exposed to influenza A virus subtype H3N2 under controlled laboratory conditions. The infected cells were subsequently fixed using 10% neutral buffered formalin to preserve cellular morphology and structural integrity prior to electrical characterization. A549 cells have been widely used as a representative human respiratory epithelial model for influenza A virus infection, including H3N2-associated host virus interaction studies [43]. In addition, formaldehyde-based fixation preserves cellular morphology through protein cross-linking and stabilizes biological structures for subsequent analytical measurements [44]. Upon receipt, the formalin-fixed H3N2-infected A549 cells were washed with PBS and serially diluted using a ten-fold dilution protocol to obtain concentrations ranging from 10^1^ to 10^6^ cells/μL. Fixed non-infected A549 cells were processed under identical conditions and diluted to the same concentration range as control samples. The infected-cell samples corresponded to H3N2 exposure levels ranging from 10^1^ to 10^6^ PFU/μL as provided by NHRI. The viral exposure level (PFU/μL) and the cell concentration (cells/μL) represent two distinct biological parameters in this study. The PFU/μL values correspond to the influenza H3N2 inoculum provided by NHRI during virus infection, whereas the cells/μL values represent the concentration of the resulting formalin-fixed A549 cell suspensions after PBS washing and serial dilution. Accordingly, the electrical measurements were performed on infected-cell suspensions with defined cell concentrations corresponding to specific viral exposure levels rather than on free virus particles. The resulting formalin-fixed H3N2-infected A549 cell suspensions (10^1^ to 10^6^ cells/μL), corresponding to viral exposure levels of 10^1^ to 10^6^ PFU/μL, together with non-infected A549 cell suspensions (10^1^ to 10^6^ cells/μL), were employed to evaluate the concentration-dependent electrical responses and screening capability of the proposed microfluidic platform.

### 2.5. Electrical Screening of H3N2-Infected A549 Cell Samples

The electrical screening procedure of the AgNPs villous-like arrays microfluidic device was connected to a source meter, and a constant bias voltage of 1 V was applied throughout the experiment. A bias voltage of 1 V was selected because it provided stable linear ohmic conduction within the investigated voltage range while minimizing unnecessary electrical loading of the AgNP-decorated sensing interface. The same operating voltage was subsequently adopted throughout all electrical measurements to ensure consistent comparison among different villous-like array geometries. For each measurement, 5 μL of formalin-fixed H3N2-infected A549 cell suspension was introduced into the sample inlet. The sample was spontaneously transported through the sensing region by capillary force without the assistance of external pumping systems. As the cell suspension traversed the AgNP-decorated villous microstructures, variations in local electrical conduction pathways generated measurable current responses, which were continuously recorded as current–time (*I–T*) signals. To evaluate concentration-dependent electrical behavior, H3N2-infected A549 cell samples with concentrations ranging from 10^1^ to 10^6^ cells/mL were examined under identical conditions, while non-infected A549 cells were used as controls. Each measurement was repeated at least five times. The resulting current variation (Δ*I* = *I* − *I_o_*), where *I* and *I_o_* represent the measured current and baseline current, respectively, together with the peak current response and signal stability, was analyzed to assess the electrical screening performance of the proposed capillary-driven microfluidic platform.

## 3. Results and Discussion

### 3.1. Compositional Characterization of AgNP-Decorated Villous-like Arrays

**Figure 3:**
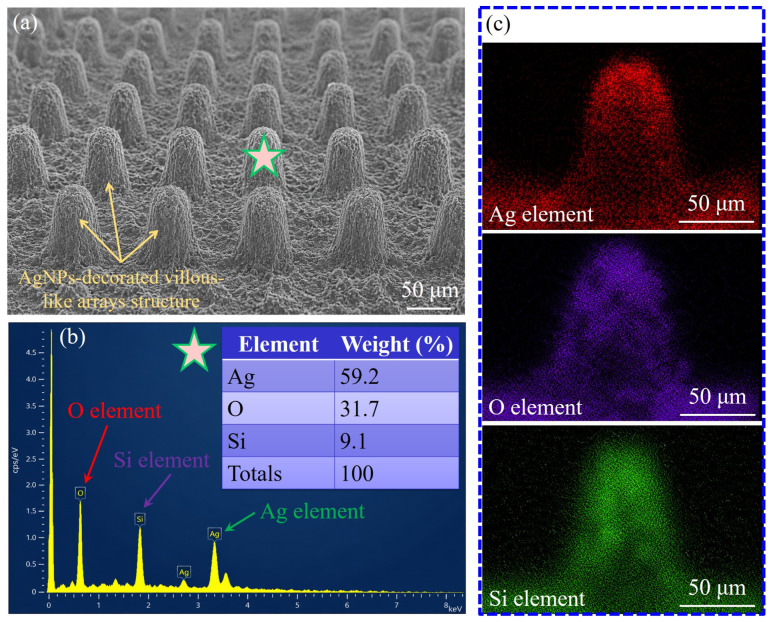
Compositional characterization of AgNP-decorated laser-patterned villous-like arrays: (**a**) SEM image of the fabricated villous-like arrays after AgNPs deposition. The star indicates the region selected for EDS analysis; (**b**) EDS spectrum and quantitative elemental composition obtained from the selected region; (**c**) EDS elemental mapping of Ag (red), O (purple), and Si (green) distributions across the villous-like microstructure.

The surface morphology, elemental composition, and elemental distribution of the AgNP-decorated laser-patterned villous-like arrays were characterized using SEM, Energy Dispersive X-ray Spectroscopy (EDS) and elemental mapping. EDS, integrated with SEM, detects characteristic X-rays generated by electron-beam interactions with the specimen, enabling qualitative and quantitative elemental analysis as well as spatial elemental mapping of the analyzed region [45], as presented in Figure 3. The SEM image (Figure 3a) revealed that the villous-like array structures retained their well-defined geometry after AgNPs deposition. The microstructures exhibited a relatively rough surface texture, indicating successful attachment of AgNPs onto both the sidewalls and top surfaces of the laser-patterned villous arrays. Such hierarchical surface features are expected to increase the effective interfacial area available for capillary transport and electrical interactions. The region marked by the star in Figure 3a corresponds to the location selected for EDS analysis presented in Figure 3b. The corresponding EDS spectrum acquired from the selected villous-like microstructure region (Figure 3b) confirmed the presence of Ag, O, and Si as the major constituent elements. Quantitative analysis revealed that Ag was the dominant element with a weight percentage of 59.2%, followed by O (31.7%) and Si (9.1%). A pronounced Ag characteristic peak was observed at approximately 3 keV, which is commonly regarded as a representative spectral signature of metallic silver and provides direct evidence of successful AgNPs deposition on the laser-patterned structures [46]. The detected O and Si signals originated primarily from the underlying soda-lime glass substrate and surface oxide species within the analyzed region. The elemental mapping results further verified the compositional distribution of the fabricated structures (Figure 3c). The Ag element exhibited continuous coverage throughout the villous-like microstructures, whereas O and Si were distributed across both the microstructures and surrounding substrate regions. Notably, the Ag signal was observed over the entire villous structure, indicating that the deposited AgNPs were successfully retained on the laser-patterned surface rather than being confined to isolated regions. The relatively uniform Ag distribution further suggests the formation of an interconnected conductive coating along the villous-like arrays. These observations confirmed the successful deposition of AgNPs onto the laser-patterned villous-like arrays and verified the formation of conductive microstructures with well-defined morphology and elemental distribution. Such characteristics are considered beneficial for establishing stable electrical conduction pathways and enhancing interfacial interactions during subsequent capillary-driven electrical measurements.

### 3.2. Structural Characteristics of AgNP-Decorated Villous-like Arrays

**Figure 4:**
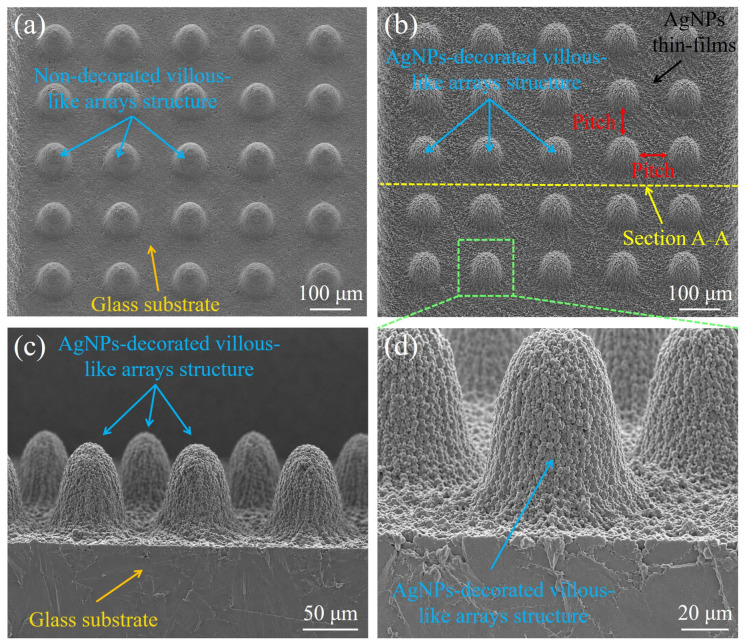
Morphological characterization of laser-patterned villous-like arrays before and after AgNPs deposition: (**a**) Top-view SEM image of non-decorated villous-like arrays on the glass substrate; (**b**) Top-view SEM image of AgNP-decorated villous-like arrays; the dashed line indicates the cross-sectional observation location (Section A–A); (**c**) Cross-sectional SEM image of the AgNP-decorated villous-like arrays formed on the glass substrate; (**d**) Higher magnification SEM image showing the hierarchical micro/nanostructured surface generated by AgNPs deposition.

To evaluate the structural characteristics of the fabricated sensing interface, the morphology of the laser-patterned villous-like arrays before and after AgNPs deposition was examined using SEM, as shown in Figure 4. The non-decorated villous-like arrays fabricated on the glass substrate are presented in Figure 4a, revealing a well-ordered arrangement of hemispherical microstructures distributed across the patterned region. After AgNPs deposition, the villous-like arrays retained their original geometry and spatial distribution, indicating that the coating process did not induce observable structural deformation (Figure 4b). The top-view SEM image of the AgNP-decorated villous-like arrays demonstrates that the deposited AgNPs formed a continuous thin-film layer covering both the villous structures and the surrounding substrate surface. The relatively uniform coating observed throughout the patterned region suggests effective diffusion and retention of the AgNPs suspension during the deposition process. Furthermore, the villous-like arrays remained clearly distinguishable after coating, preserving the designed interpillar pitch and microstructure arrangement required for capillary-guided sample transport. The cross-sectional SEM image shown in Figure 4c further confirms the successful formation of three-dimensional villous-like microstructures on the glass substrate. The structures exhibited a height of approximately 80 μm and maintained a stable attachment to the underlying substrate. A conformal AgNPs coating layer was observed along the sidewalls and top surfaces of the villous structures, indicating effective nanoparticle coverage over the entire microstructured region. A higher magnification SEM image of the selected region (Figure 4d) revealed that the villous surface was composed of densely packed AgNP aggregates, resulting in a hierarchical micro/nanostructured morphology. Such surface roughening substantially increased the effective interfacial area and provided a larger conductive contact region for subsequent sample interaction. The combination of uniformly distributed villous-like arrays and continuous AgNP conductive coating is expected to facilitate capillary-driven transport and improve electrical signal transduction within the proposed microfluidic platform [47].

### 3.3. Wettability and Capillary Transport Characteristics of AgNP-Decorated Villous-like Arrays

**Figure 5:**
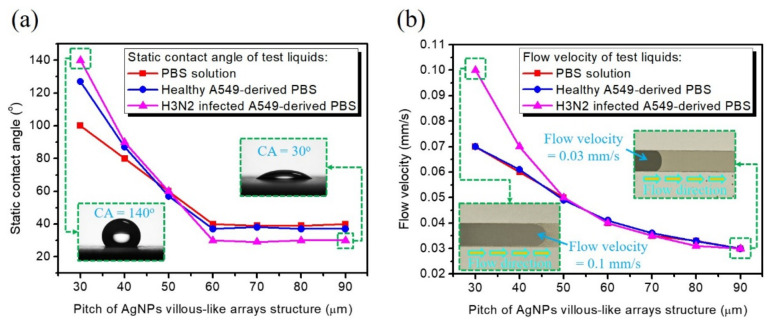
Wettability and capillary transport behavior of AgNP-decorated villous-like arrays with pitches ranging from 30 to 90 μm: (**a**) Static contact angles of PBS solution, healthy A549-derived PBS, and H3N2-infected A549-derived PBS. Insets show representative CA of 140° and 30°; (**b**) Capillary-driven flow velocities of the corresponding liquids. Insets show representative flow behaviors at 0.1 mm/s and 0.03 mm/s.

The wettability and capillary transport characteristics of the AgNP-decorated villous-like arrays were evaluated using PBS solution, healthy A549-derived PBS, and H3N2-infected A549-derived PBS, as shown in Figure 5. The static contact angle (CA) measurements revealed a strong dependence on the interpillar pitch of the villous-like arrays (Figure 5a). For all tested liquids, the apparent contact angle decreased markedly as the pitch increased from 30 μm to 90 μm. The H3N2-infected A549-derived PBS exhibited the highest contact angle of 140° at a pitch of 30 μm, followed by healthy A549-derived PBS (127°) and PBS solution (100°). As the pitch increased beyond 60 μm, the CA of all test liquids gradually decreased and stabilized within the range of 30° to 40°. The pronounced hydrophobic behavior observed at small pitch values can be attributed to the increased fraction of trapped air pockets between adjacent villous structures, resulting in a composite solid–liquid–air interface [48]. Such behavior is characteristic of a Cassie–Baxter dominant wetting state. As the interpillar spacing increased, liquid penetration into the microstructured surface became more favorable, increasing the effective solid–liquid contact area and gradually shifting the wetting regime toward a Wenzel state [49]. Similar wetting transitions have been reported in laser-patterned hierarchical surfaces, where both microscale geometry and nanoscale surface roughness contribute to the apparent contact angle behavior [50].

A fixed sample volume of 5 μL was introduced into the inlet reservoir, and the movement of the liquid front was recorded using an optical imaging system. The average capillary flow velocity was determined by dividing the transport distance by the elapsed filling time. The capillary-driven flow proceeded until the sensing microchannel became completely filled, after which the flow naturally ceased as capillary equilibrium was reached [23]. The capillary-driven flow behavior exhibited a trend consistent with the wettability results (Figure 5b). The flow velocity decreased with increasing interpillar pitch for all tested liquids. At an interpillar pitch of 30 μm, the H3N2-infected A549-derived PBS exhibited the highest flow velocity (0.1 mm/s), whereas both healthy A549-derived PBS and PBS exhibited flow velocities of 0.07 mm/s, corresponding to a relative difference of 0.03 mm/s (30%). As the interpillar pitch increased to 40 μm, the flow velocities decreased to 0.07, 0.061, and 0.06 mm/s for H3N2-infected A549-derived PBS, healthy A549-derived PBS, and PBS, respectively, reducing the maximum difference to 0.01 mm/s (14.3%). At an interpillar pitch of 50 μm, the measured flow velocities further converged to 0.05, 0.049, and 0.05 mm/s, with a maximum difference of only 0.001 mm/s (<2%). Similar convergence was observed at larger interpillar pitches. At 60 μm, the flow velocities were 0.04, 0.041, and 0.04 mm/s, corresponding to a difference of 0.001 mm/s (<2.5%). At 70 μm, the three liquids exhibited flow velocities of 0.035, 0.036, and 0.035 mm/s, with a maximum difference of 0.001 mm/s (<2.9%). The differences became even smaller at 80 μm (0.033, 0.0329, and 0.031 mm/s), corresponding to a maximum variation of 0.002 mm/s (<6.5%), whereas identical flow velocities of 0.03 mm/s were obtained for all three liquids at 90 μm. These quantitative results indicate that the influence of liquid composition on capillary transport progressively diminished with increasing interpillar spacing, whereas the microstructure geometry became the dominant factor governing passive capillary flow. Nevertheless, the H3N2-infected A549-derived PBS consistently exhibited the highest flow velocity throughout the investigated pitch range. The enhanced flow velocity observed in densely packed villous-like arrays can be attributed to the increased capillary pressure generated within the confined microstructured spacing. The combination of AgNP-induced nanoscale roughness and laser-patterned microscale villous structures therefore provided a cooperative mechanism for regulating both surface wettability and passive liquid transport. Such hierarchical capillary interfaces have been reported to improve liquid-handling efficiency, sample utilization, and operational stability in self-driven microfluidic sensing systems [51].

### 3.4. Electrical Response Characteristics of AgNP-Decorated Villous-like Arrays

**Figure 6:**
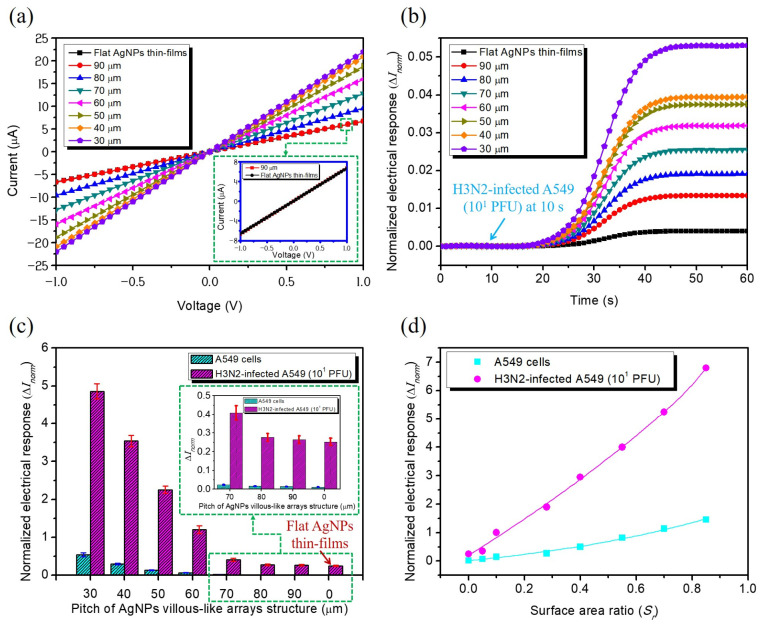
Electrical characterization of AgNP-decorated villous-like arrays with different interpillar pitches: (**a**) *I–V* characteristics of villous-like array electrodes with pitches ranging from 30 to 90 μm and a flat AgNP thin-film electrode; (**b**) Normalized *I–T* responses following the introduction of H3N2-infected A549 cell samples (10^1^ PFU) at 10 s; (**c**) Comparison of normalized electrical responses between H3N2-infected A549 cells (10^1^ PFU) and non-infected A549 cells as a function of interpillar pitch. Insets show enlarged views of the responses at pitches ranging from 70 to 90 μm and the flat AgNP thin-film electrode; (**d**) Relationship between normalized electrical response and surface area ratio for H3N2-infected and non-infected A549 cell samples. Data are presented as mean ± standard deviation (SD) obtained from five independent measurements (n = 5). Error bars represent the standard deviation of the repeated measurements.

The electrical characteristics of the AgNP-decorated villous-like arrays were investigated using H3N2-infected A549 cell samples within the capillary-driven microfluidic platform, as shown in Figure 6. The current–voltage (*I–V*) characteristics of devices with interpillar pitches ranging from 30 μm to 90 μm were compared with those of a flat AgNPs thin-film electrode without villous structures (Figure 6a). Under an applied voltage range from −1 V to 1 V, all devices exhibited linear and symmetric current responses, indicating stable ohmic conduction behavior. At an applied voltage of 1 V, the measured currents increased progressively with decreasing interpillar pitch. The device with a 30 μm pitch exhibited the highest current response of 22 μA, whereas the flat AgNPs thin-film electrode showed the lowest current response of 6.65 μA. The enhanced electrical conductivity observed for densely packed villous-like arrays is attributed to the increased effective conductive surface area and the formation of interconnected AgNPs conductive pathways, which facilitate charge transport across the sensing interface [52]. The transient electrical responses of the fabricated devices were further evaluated using H3N2-infected A549 cell samples corresponding to a viral exposure level of 10^1^ PFU (Figure 6b). The infected-cell sample was introduced into the microfluidic channel at 10 s, and the normalized electrical response (${\Delta I}_{norm}$) was calculated according to:

(1) $${\Delta I}_{norm}=|\frac{I-I_{0}}{I_{0}}|$$

where *I* denotes the measured current after sample introduction and *I_0_* denotes the baseline current prior to sample loading. Δ*I_norm_* represents the relative current variation and provides a dimensionless parameter for comparing the electrical screening performance among different villous-like array geometries. Following sample introduction, all villous-like array electrodes exhibited a rapid increase in electrical response before reaching a stable plateau. The Δ*I_norm_* increased from 0.004 for the flat AgNPs thin-film electrode to 0.053 for the 30 μm pitch structure. This enhancement is attributed to the enlarged effective electrode–sample interaction area and improved interfacial charge-transfer behavior provided by the villous-like microstructures [17].

The influence of structural geometry on sensing performance was further examined using the surface area ratio (*S_r_*), where *S* represents the effective surface area of the AgNP-decorated villous structures and *S_o_* denotes the projected planar area of the sensing region:

(2) $$S_{r}=\frac{S}{S_{0}}$$

As shown in Figure 6c, the normalized electrical response of H3N2-infected A549 cells increased markedly as the interpillar pitch decreased from 90 μm to 30 μm. Specifically, the normalized response increased from 0.264 at a pitch of 90 μm to 4.86 at a pitch of 30 μm. In contrast, non-infected A549 cells exhibited only minor signal variations, with Δ*I_norm_* increasing from 0.0143 to 0.539 over the same pitch range. The strongest response was consistently obtained from the 30 μm pitch structure, whereas the flat AgNPs thin-film electrode exhibited the lowest response for both sample groups. The relationship between normalized electrical response and surface area ratio is presented in Figure 6d. For H3N2-infected A549 cells, the Δ*I_norm_* increased from 0.25 to 6.8 as the *S_r_* increased from 0 to 0.85. By comparison, the response of non-infected A549 cells increased from 0.02 to 1.46 over the same range. More importantly, the H3N2-infected A549 samples exhibited electrical responses that were approximately 4.7-fold higher than those of the control group at the maximum surface area ratio. These results indicate that the enlarged effective surface area provided by the villous-like arrays significantly increased cell-electrode interaction probability and amplified local electrical perturbations during capillary-driven transport. Similar improvements in signal transduction resulting from hierarchical micro/nanostructured conductive interfaces have been reported in electrochemical and electrical biosensing platforms [53,54,55]. The error bars shown in Figure 6 represent the standard deviation obtained from repeated independent measurements, demonstrating good measurement repeatability and consistent electrical responses for each experimental group.

### 3.5. Electrical Screening of H3N2-Infected A549 Cells Using AgNP-Decorated Villous-like Arrays

**Figure 7:**
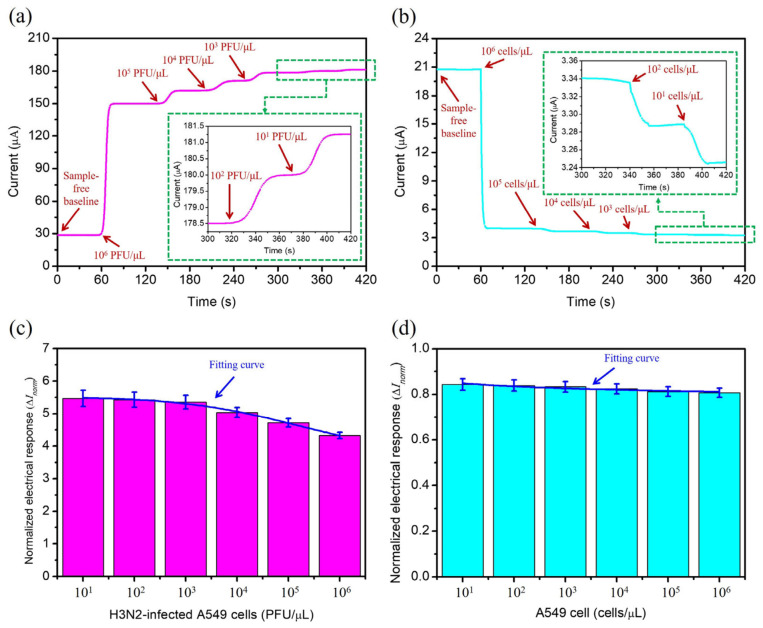
Electrical screening performance of H3N2-infected A549 cells using the capillary-driven microfluidic platform: (**a**) *I–T* responses of H3N2-infected A549 cells with viral exposure levels ranging from 10^6^ to 10^1^ PFU/μL. Inset shows enlarged responses at low viral exposure levels (10^2^–10^1^ PFU/μL); (**b**) *I–T* responses of non-infected A549 cells with concentrations ranging from 10^6^ to 10^1^ cells/μL. Inset shows enlarged responses at low cell concentrations (10^2^–10^1^ cells/μL). (**c**) Normalized electrical responses of H3N2-infected A549 cells as a function of viral exposure level. (**d**) Normalized electrical responses of non-infected A549 cells as a function of cell concentration. Error bars represent the standard deviation of repeated measurements, and solid lines indicate fitting curves.

The electrical screening performance of the capillary-driven microfluidic platform was evaluated using formalin-fixed H3N2-infected A549 cells and non-infected A549 cells, as shown in Figure 7. All measurements were conducted at an applied voltage of 1 V, and the baseline current prior to sample introduction was used as the reference signal. Representative current–time (*I*−*T*) responses of H3N2-infected A549 cells with viral exposure levels ranging from 10^6^ to 10^1^ PFU/μL are shown in Figure 7a. Following sample introduction, stepwise increases in current were observed, and the current increased from 28.5 μA to 181.2 μA. Enlarged views further demonstrated distinguishable responses between 10^2^ and 10^1^ PFU/μL, indicating the capability of the platform to resolve low-level infected-cell samples. In contrast, non-infected A549 cells exhibited an opposite response trend (Figure 7b). As the concentration decreased from 10^6^ to 10^1^ cells/μL, the current decreased from 20.75 μA to 3.25 μA. Although concentration-dependent variations were observed, the signal magnitude remained substantially lower than that of H3N2-infected A549 cells, indicating distinct electrical responses between infected and non-infected samples [56].

The normalized electrical responses are summarized in Figure 7c,d. H3N2-infected A549 cells exhibited Δ*I_norm_* of 5.47, 5.43, 5.36, 5.04, 4.71 and 4.32 for viral exposure levels ranging from 10^1^ to 10^6^ PFU/μL, respectively. By comparison, non-infected A549 cells showed only minor variation, decreasing from 0.84 to 0.81 over the same concentration range. The small error bars observed in both datasets further confirmed the repeatability of the electrical measurements. In summary, the AgNP-decorated villous-like arrays enabled clear electrical discrimination between H3N2-infected A549 cells and non-infected A549 cells over the investigated concentration range. The combination of capillary-driven sample transport and hierarchical conductive microstructures generated concentration-dependent signal variations and enhanced electrode–sample interactions, demonstrating the feasibility of the proposed platform as a label-free electrical screening strategy for virus-associated cellular samples [54,57].

To further position the proposed platform among recent influenza-related biosensing technologies, representative sensing systems are compared in Table 1 [58,59,60,61,62]. Previous studies have achieved high analytical sensitivity through electrochemical, paper-based microfluidic and chemiluminescent sensing approaches. However, many of these platforms rely on target-specific recognition elements, signal amplification processes, or additional instrumentation. In contrast, the proposed platform enables direct electrical screening of H3N2-infected A549 cells using AgNP-decorated laser-patterned villous microstructures integrated within a capillary-driven microfluidic device. By combining passive capillary transport and conductive microstructured electrodes on a single glass substrate, the device operates without external pumping, fluorescence labeling, or molecular amplification. Overall, although several reported systems provide lower detection limits for purified viral proteins, nucleic acids, or biomarkers, the present approach emphasizes structural integration, self-driven operation, and label-free electrical sensing of virus-associated cellular samples. These features demonstrate the feasibility of the proposed platform for rapid microfluidic electrical screening applications.

Although the present study demonstrates good measurement reproducibility under identical fabrication and testing conditions, systematic evaluations of long-term operational stability, storage stability, and batch-to-batch fabrication consistency were beyond the scope of this work. As highlighted in recent studies on electrochemical biosensor integration, these factors remain essential for future practical deployment and commercialization of biosensing platforms [63]. Future work will therefore focus on evaluating these aspects under extended operating and storage conditions. Another limitation of the present study is that the electrical screening experiments were performed using formalin-fixed H3N2-infected A549 cells rather than freshly isolated clinical specimens. Formalin fixation is widely employed to preserve cellular morphology and improve biosafety through protein cross-linking; however, it may also alter membrane permeability and cell–electrode interfacial electrical characteristics, potentially influencing the measured electrical responses [64]. Future studies should therefore include validation using fresh clinical specimens, different influenza virus strains, and larger biological sample populations to further assess the clinical applicability and robustness of the proposed electrical screening platform, consistent with recent perspectives on electrochemical cell-based biosensors.

## 4. Conclusions

A capillary-driven microfluidic electrical screening platform based on AgNP-decorated laser-patterned villous-like arrays was developed for the analysis of H3N2-infected A549 cells. Hierarchical conductive microstructures fabricated on glass exhibited tunable wettability and capillary transport characteristics, with CA ranging from 140° to 30° and flow velocities ranging from 0.1 to 0.03 mm/s as the interpillar pitch increased from 30 to 90 μm. Among the tested structures, the 30 μm pitch arrays produced the highest current response of 22 μA and the largest Δ*I_norm_* of 0.053. The platform enabled electrical differentiation between H3N2-infected and non-infected A549 cells over a viral exposure range of 10^1^–10^6^ PFU/μL, with Δ*I_norm_* of 5.47–4.32 and 0.84–0.81, respectively. These findings demonstrate that AgNP-decorated villous-like arrays can effectively enhance capillary-driven electrical sensing without external pumping, fluorescence labeling, or molecular amplification. The proposed platform provides a simple and fabrication-oriented strategy for rapid electrical screening of virus-associated cellular samples and offers potential for future self-driven microfluidic biosensing applications.

## Figures and Tables

**Table 1:** Comparison of representative influenza-related biosensing platforms and the proposed capillary-driven AgNPs microfluidic electrical screening device.

Fabrication Method	Material	Target	Limit of Detection	Reference
AgNP-decorated laser-patterned villous microstructures	AgNPs/glass substrate	H3N2-infected A549 cells	10^1^ PFU/μL	This study
Label-free electrochemical peptide sensor	Porous BSA/MXene nanocomposite/streptavidin-modified Au electrode	Influenza H5N1 neuraminidase protein	0.098 nM	Kim et al. (2023) [58]
Sequential-injection paper-based microfluidics with E-RT-LAMP	Paper microfluidic chip/methylene blue redox probe	Influenza A virus RNA	0.152 pM	Lima et al. (2026) [59]
Electrochemical biosensor integrated with airborne sampling interface	Comb-shaped capacitive Au electrode/SAM of SH-biotin/streptavidin/biotinylated aptamer	H1N1 hemagglutinin protein	0.045 pg/mL	Chen et al. (2025) [60]
Microfluidic paper-based chemiluminescence sensing platform	PEI/CaCO_3_-functionalized μPAD chemiluminescence interface	Avian influenza virus biomarkers	0.32 pM	Tian et al. (2024) [61]
Allosteric probe-initiated dual rolling circle amplification biosensor	Functional DNA probe/RCA amplification interface	*E. coli* O157:H7	1.6 CFU/mL	Sun et al. (2024) [62]

## Data Availability

All the data generated or analyzed during this investigation are included in this article.
